# Supplementary material for: Are carbon emissions trading and green financial instruments synergistic? -Comprehensive quantitative research based on content analysis
Source: PLoS One. 2024 Mar 7;19(3):e0298601. doi: 10.1371/journal.pone.0298601 (PMC10919601; doi:10.1371/journal.pone.0298601)
Supplement: S1 Data — (ZIP) [file pone.0298601.s001.zip › Data/地级市Z_F工作报告.zip!/╔╜╢1⁄2/╝├─■╩╨/└┤╫╘╣1⁄2╓┌║┼í╛┬φ┐╦╩2╛▌═°í┐_2005.docx]

各位代表：

　　现在，我代表济宁市人民政府向大会报告工作，请予审议，并请市政协委员和其他列席会议的同志提出意见。

　　一、2004年经济社会发展取得新成绩

　　过去的一年，在中共济宁市委的正确领导下，经过全市人民的共同努力，圆满完成市十四届人大二次会议确定的各项任务目标，“十五”计划主要指标提前一年完成，在全面建设小康社会进程中迈出坚实的一步。

　　（一）经济发展跃上新台阶。全市地区生产总值完成1102亿元、增长17.2%，三次产业比例由上年的14.4∶49∶36.6调整为13.8∶51.6∶34.6。水利等农业基础设施建设步伐加快。农业“五大产业链”建设成效明显，粮食总产346万吨、增长6.8%，粮食安全得到保障。林牧渔业全面增长。农村供销合作组织活力增强。农业机械化水平稳步提高。新增农村劳动力转移就业12万人。全市规模以上工业企业达到1078家、新增309家，利税过千万元企业129家、新增26家。高新技术产业比重提高到19%。新创“山推”、“英克莱”两个中国驰名商标。社会消费品零售总额293.8亿元、增长16.5%，进出口总额14.44亿美元，其中出口8.25亿美元、增长54.7%。相继引入南京中央百货、济南贵和等知名商业品牌，国有商业企业改制重组实现新突破。我市跨入中国优秀旅游城市行列，旅游社会收入62.69亿元、增长72.7%。全社会固定资产投资508亿元、增长42.7%，实际利用外资4.12亿美元、增长105%。向国家、省争取土地复垦、道路交通及航运、水利建设专项资金13.3亿元。土地市场秩序治理整顿取得实效。开工建设500万元以上项目1300个，其中亿元以上项目133个，嘉达纺织、陆通轮胎一期等577个项目竣工投产。对区域经济发展具有深远影响的中国重汽商用车项目开工建设。全市财政总收入104.7亿元，其中地方财政收入54.06亿元、增长24.4%，税收收入占地方财政收入的比重达到73.1%。争取用活财税政策，新增地方财政收入2亿多元。民营经济上缴税金39.92亿元、增长45.3%。金融机构年末存款余额778.9亿元，贷款余额543.8亿元，分别比年初增加115.4亿元、72.8亿元。

　　（二）城乡人居环境日益优化。市区“绿亮清”工程当年完成投资37.5亿元。济宁至曲阜快速干道建成通车，日东高速公路济宁城区入口贯通，共青团路等23条主干道和20条小街巷建设改造任务完成，新增公共绿地71万平方米。粮贸大厦和康乐中心“半拉工程”启动盘活。清除违法建筑、乱贴乱画和防治二氧化硫低空污染及市区河道水循环工程取得阶段性成效。机动车禁鸣范围扩大。7处城市污水处理厂投入运行或试运行。15家电厂完成脱硫，化工发酵企业异味污染得到遏制，城区空气质量优良天数由上年的280天增加到335天。县城及镇村规划建设增添新亮点。济宁至北京始发临客列车运行良好。

　　（三）各项社会事业获得进步。完成重要科技成果156项，其中25项荣获省科学技术奖。申报专利523件。第五届中国专利高新技术产品博览会和“院士济宁行”等项科技交流与招商活动成效显著，引进新技术、新成果324项和急需人才1500名，新建产学研基地10处。济宁市生产力促进中心被认定为国家级示范中心。完成中小学校舍危房改造5.5万平方米，高中扩招新生8636人，投资2.4亿元、建筑面积18万平方米的济宁学院一期工程竣工。成功举办国际孔子文化节和世界旅游日中国主会场活动及中国京杭运河文化节，一批文艺精品在全国全省获奖。公共卫生“两大体系”建设进度加快。有效预防高致病性禽流感疫情发生。完成省下达人口与计划生育年度责任目标，人口自然增长率为5.96‰。双拥共建、优抚安置、国防教育、民兵预备役建设得到加强。新闻出版、广播电视、外事侨务、民族宗教、地震气象、体育、档案、史志、文博及对台、人防、老龄、残疾人等项事业获得新的发展。

　　（四）人民生活水平不断提高。全市城镇居民人均可支配收入9502元、增长14.3%，农民人均纯收入3648元、增长14.8%。新增城镇就业10.08万人，其中下岗失业人员再就业4.76万人，城镇登记失业率为3.4%。企业养老保险参保人数达到44.78万人，新增3万人，职工医疗保险和工伤保险参保人数分别达到50.4万人、27.6万人。各级财政投入城乡低保资金4580万元，基本实现应保尽保、应助尽助。12352名特困生得到救助。省、市财政转移支付资金按计划拨付到位，将6个经济薄弱县的资源税收入全额纳入县乡财政盘子。落实“三补一降”支农扶持政策，全市农民人均受益71.5元。清欠农民工工资7394万元。累计完成农村改水改厕114.13万户，农村合作医疗试点人口扩大到70.5万人。新增“村村通油路”里程3400公里，行政村通客车率达到98.8%。3.6万平方米经济适用房全面竣工，市区302户困难居民迁入廉租房。年初承诺办好10件实事如期完成。

　　（五）民主法制和精神文明建设进一步加强。认真执行人大及其常委会决议决定，支持人民政协参政议政，办复人大代表建议152件、政协委员提案342件。积极贯彻《行政许可法》，确定79个市级行政许可实施主体，市行政审批服务中心工作经验在全省推广。严格执行《统计法》、《审计法》。加强廉政建设，提高拒腐防变能力。文明城市创建广泛深入，精神文明建设取得新成果。“平安济宁”建设扎实推进，安全生产形势总体平稳。

　　各位代表!过去一年全市经济社会发展取得的辉煌成就，是省委、省政府和市委科学决策、正确领导，市人大有效监督、大力支持，市政协参政议政、民主监督的结果，是全市干部群众团结奋斗、开拓创新的结果。在此，我代表市人民政府，向全市广大工人、农民、知识分子和干部，向各民主党派、工商联、人民团体和各界人士，向中国人民解放军驻济部队指战员、武警官兵和公安干警，向中央、省驻济单位，向来济宁创业发展的各位投资者和建设者，向关心支持济宁现代化建设的港澳台同胞、海外侨胞和国际友人，表示衷心感谢，致以崇高敬意！

　　我们也清醒地看到，我市经济社会发展还存在一些差距和不足，主要是：经济结构层次不高，服务业发展不快，对外经贸和高新技术产业比重偏低，规模以上工业企业数量少，市属企业改革进展缓慢；县域经济发展不平衡，部分县乡财源匮乏，可用财力缺口较大；农业和农村经济结构调整深度不够，农民持续增收的基础不够稳固；就业供需矛盾突出，社会保障覆盖面不宽，部分群众生活困难；社会事业发展存在薄弱环节，人口资源环境面临较大压力。政府执政能力和领导水平还不能很好适应新形势新任务要求,少数干部观念滞后,作风不实,标准不高,要求不严,影响发展的问题时有发生。廉政建设监督不力，管理不严，个别干部堕落犯罪。对这些问题，我们将采取有效措施，认真加以整改。

　　二、新一年政府工作总体要求和任务目标

　　根据省委、省政府和市委的部署安排，今年政府工作总体要求是：高举邓小平理论和“三个代表”重要思想伟大旗帜，深入贯彻党的十六大和十六届三中、四中全会精神，以科学发展观统揽全局，围绕建设经济强市、文化名市、组群结构大城市目标，把握解放思想、干事创业、比学赶超、加快发展总基调，突出抓好扩大开放、深化改革、调整结构、科技创新、工业立市、民营经济、旅游产业、生态城市八项重点，努力做到农民增收、就业增加、财政增长、社保增强，着力构建和谐社会，促进全市经济社会全面协调可持续发展。年度主要预期目标是：地区生产总值增长14%，地方财政收入增长17%，全社会固定资产投资增长30%，外贸进出口增长20%，其中出口增长20%，实际利用外资增长35%，社会消费品零售总额增长14%，城镇居民人均可支配收入增长8%，农民人均纯收入增长5%，城镇登记失业率控制在4%以内，人口自然增长率控制在6.8‰以内。

　　完成今年各项任务目标，面临诸多机遇和有利条件。我国经济正处在新一轮增长周期的上升期，市场经济体制日趋完善，经济国际化进程明显加快，改革开放进入新的阶段，为我们加快发展提供了良好宏观环境。我市煤电水资源得天独厚，具备承接国内外产业、资本转移的独特优势；已经投产和正在建设的一批产业项目，成为拉动区域经济的重要增长点；一系列改革调整措施进一步完善落实，经济增长的内生动力持续增强，为我们加快发展提供了有力支撑。全市各级领导班子把握发展大势、驾驭市场经济的能力不断提高，干部群众风正气顺心齐劲足，比学赶超、干事创业的氛围更加浓厚，为我们加快发展提供了可靠保障。同时也要看到，国际经济增长存在许多不确定因素，国家宏观调控力度继续加大，区域经济竞争日益加剧，给我们加快发展带来一些新情况、新问题。我们要切实增强机遇意识、忧患意识、创新意识，调动和发挥方方面面的积极性、主动性、创造性。坚持把科学发展、加快发展作为第一要务，全力争取有质量、有效益、有后劲的速度，实现经济社会既快又好发展。坚持把以人为本、执政为民作为第一责任，努力实现经济发展与造福人民的协调统一。坚持把求真务实、真抓实干作为第一要求，不图虚名，埋头苦干，创造经得起检验的业绩，谋求富有后劲的发展，不断开创全市改革开放和现代化建设的新局面。

　　三、努力提高经济增长质量和效益

　　以改革为动力，以调整为主线，以开放为重点，加快转变经济增长方式，增创竞争发展新优势。

　　(一)强力推进对外开放。进一步健全完善政府为主导、企业为主体的外经贸工作机制，创新招商方式，注重招商实效，调整出口结构，扩大出口规模，实现外向型经济发展新突破。

　　打造招商平台。强化省批园区招商引资的龙头作用。济宁高新区和曲阜新区立足优化园区要素配置和产业布局，重点发展与国内外大公司、大商社及其研发机构的合资合作，着力引进“高大外”项目，尽快形成外向型产业集群。强化节会招商引资的集聚功能。突出经贸合作，完善市场运作，创新国际孔子文化节办节方式，把握会前广泛洽谈、会上集中推进、会后跟踪落实三个环节，提高外资项目成功率。强化“中国孔子经贸网”招商引资的窗口效应。实现与国际国内知名网站的链接，有效开展网上洽谈和项目初审。强化企业招商引资的主体作用。引导各类企业特别是大企业走向开放第一线，谋求合资合作，实现强强联合。借鉴山推国际事业园“以商招商”成功模式，突出抓好产业链招商，壮大外资企业群体。指导外贸企业与国外贸易伙伴联手创办外资项目，实现向“三资”企业的转变。进一步拓展招商领域，力争在服务业和公共事业利用外资上取得突破。

　　整合招商队伍。面向海内外选聘高层次外经贸人才，充实专业招商力量，派驻重点国家和地区蹲点招商。强化对各类招商代理激励约束，扩大代理招商成果。明确各类出国团组招商引资责任，增强出访活动的目的性、实效性。加强全市招商力量、经贸信息、项目资源的有机整合和调度协调，提高对外开放整体效益。

　　优化招商环境。积极接轨国际惯例和通行规则，为外商提供优质高效的行政服务。全面推行外资项目审批“代办制”，建立项目建设协调联席会制度，及时解决问题，切实兑现承诺。在全社会营造亲商、安商的浓厚氛围，最大限度地为外商工作生活提供便利条件，严肃查处侵害投资者合法权益的行为。

　　扩大对外贸易。调整优化出口商品结构，提高出口产品的科技含量和附加值。培植出口创汇骨干企业。大力发展加工贸易。积极应对国际贸易壁垒，切实保证出口企业利益。完善海关、检验检疫服务网络，建立进出口货物“绿色通道”。支持有实力的企业发展境外带料加工、工程承包和劳务合作。

　　(二)做大做强工业经济。继续实施“工业立市”战略，坚持改革调整与科技进步并重，扩张总量与产业升级齐抓，加快构筑产业结构优化、规模效益突出、竞争优势明显、发展后劲充足的工业体系。

　　提升壮大一批主导产业。有效实施煤炭产业战略转型，推广“一矿一区”模式，大力兴办煤炭精深加工和新型制造业项目。加快凯赛生物技术产品系列开发和菱花正大赖氨酸项目建设，促进生物技术产业做大做强。壮大汽车和工程机械生产规模，增加整机品种，发展配套项目，拉长产业链条，提高产业集中度。以鲁抗、雪花集团等为载体，引进前沿技术加快开发医药新产品，增创医药食品产业竞争发展新优势。更好地服务于嘉达纺织、德源纱厂投产达产，放大如意、樱花品牌效应，促进纺织服装产业整体上水平。扩大高标号水泥、高档机制纸生产能力，提高建材及造纸产业科技含量。

　　扩张发展一批骨干企业。通过招商引资、资产重组、科技创新和发展民营经济等途径，大幅增加规模以上企业数量。全市规模以上工业企业突破1500家。采取外资嫁接、资本运营、挂强联名等措施，促使现有骨干企业晋档升级。全市利税过千万元企业发展到160家，新增31家。选择一批主业突出、成长强劲的企业，加快实施“创百亿工程”。今年力争销售收入过10亿元企业达到28家，新增10家，其中4家企业突破50亿元，为2007年6至8家企业过百亿奠定基础。

　　引进建设一批重点项目。强化包保责任，优化建设环境，集中抓好976个500万元以上产业项目建设。加快501个续建项目建设进度，力争年内投产率达到60%以上。开工建设475个新项目，力争当年竣工投产率达到45%。组成专门班子跟踪服务，确保完成191个亿元以上大项目年度建设任务。

　　培育开发一批拳头产品。按照“主导产品龙头化、龙头产品规模化”的要求，选择优势产品实施重点培育，年内力争单个产品销售收入过亿元的超过50个。坚持引进转化与自主研发相结合，加快高新技术产品开发，力争填补我市高新技术产业的行业空白，高新技术产业比重提高2个百分点。强化企业商标品牌意识，引导有条件的企业争创中国驰名商标和省著名商标。

　　(三)深度调整农业结构。把调整结构作为解决“三农”问题的关键举措，制定落实更直接、更有效的政策措施，培育拉长“五大产业链”，加快农业产业化、标准化、现代化进程，促进农业增效、农民增收、农村经济全面发展。

　　抓好典型引导调整。推出一批农副产品种养加工典型，通过现身说法、良种良法，带起连片调整，发展一批专业村。结合区域化布局，发挥专业村辐射效应，建设一批专业镇和产业带。

　　建设基地促进调整。坚持因地制宜、发挥优势，依托现有产业基础，高起点、高标准规划建设一批农副产品基地，提升种养业规模档次。加快建设无规定动物疫病区，建立健全农产品质量标准体系、检验检测体系和认证体系，促进特色基地向无公害、绿色、有机食品基地迈进。

　　培植龙头带动调整。鼓励引导民间资本和外商资本兴办农副产品精深加工项目，支持销售收入过亿元骨干龙头企业扩量提质，增强辐射带动能力。积极发展农村合作经济组织。密切龙头企业与基地、农户利益联结，推行“订单农业”，发展“绿色车间”。

　　完善服务保障调整。多渠道增加农业投入，加大财政支农力度，积极发展农户小额信贷和联保贷款。加快交通、通讯、电力、水利等基础设施网络向农村特别是产业基地延伸覆盖。年内完成2.1万项农田水利建设项目。大力推广实用技术，及时为农民提供农产品供求信息，办好各类农业科技示范园区。加强产地市场建设，开辟农产品“绿色通道”。抓好劳动技能培训和输出基地开发，年内新增农村劳动力转移就业20万人。

　　落实政策推动调整。今年农业税税率再减征2个百分点。继续兑现对种粮农民直补、良种补贴和购置大型农机具补贴等项政策。整顿规范各种涉农收费。切实保护基本农田，稳定和完善农村土地承包关系，引导和规范土地流转。

　　(四)创新发展旅游产业。以荣获中国优秀旅游城市为新起点，进一步完善规划，理顺关系，创新机制，突出抓好“三个开发”。一是抓好旅游产品开发。在依法确保文物安全、保护自然生态的前提下，探索建立旅游行政管理部门与主体旅游资源管理机构相统一的新型旅游管理体制，加快旅游管理、开发、经营一体化进程。集中抓好主要景区景点综合开发，提升旅游主导产品档次水平。强化“三孔”龙头带动，整合景区景点，面向海内外游客，设计推出品位高、体量大、代表济宁旅游整体形象的精品线路；搞好市内景区景点衔接，面向城乡群众，开辟一批各具特色的短距线路。二是抓好服务功能开发。配套完善景区服务设施，招商建设高档旅游接待场所，加强旅游从业人员培训，规范服务标准与程序，搞好旅游市场秩序综合整治。开发健康、文明的娱乐项目，延长游客游览时间。创新旅游企业经营机制，组建新型旅游企业集团。三是抓好客源市场开发。借助主流媒体，宣传推介济宁旅游产品。打响孔子品牌，深入重点客源市场促销。积极发展与国内外大型旅游集团、旅行社联合协作，结合全省无障碍旅游区建设，加强与周边旅游城市协作联动，力争在市场双向开拓、客源共享方面取得实效。

　　发挥旅游业综合带动效应，促进服务业全面发展。继续引入品牌优势突出、核心竞争力强的国内外大型流通企业，参与我市商贸企业改制重组。积极发展连锁、配送等新型业态，培植壮大现代物流企业。大力兴办中介咨询、社区服务、物业管理等新兴三产项目。强化工商行政管理和市场物价监管，高标准建设“鲁南质量检测中心”。健全社会信用体系，推进金融安全区建设，着力打造“诚信济宁”。

　　(五)继续深化各项改革。深化国有资产监管体制改革，建立国有资产出资人制度和监督管理体系。推进各类企业规范化股份制改造，健全法人治理结构，建立现代企业制度。加快市属企业改制进程，突出抓好困难企业脱困，严格规范破产操作，妥善安排职工生产生活。稳步推进事业单位综合配套改革，精简财政供养人员，改革用人和分配制度。立足建立公共财政体制框架，积极推行部门预算，完善国库集中支付制度。支持金融企业深化改革，继续推进行政审批制度、粮食流通体制和户籍制度等项改革。

　　(六)努力壮大县域经济。继续实施促强扶弱战略，鼓励经济强市扩大领先优势，不断进位升级。支持经济薄弱县采取更加务实的政策，发挥比较优势，实现跨越发展。完善“双30”乡镇动态管理，促进乡镇经济竞相发展。进一步健全完善县域经济社会发展综合评价和考核体系，调动和发挥好县乡科学发展、加快发展的积极性。把振兴工业作为县域经济发展的主攻方向。持续增加有效投入，引进建设创税项目；推进现有企业改组改造，促其增强活力、扩张规模，各县市区全年新增投资过千万元项目30个以上，新增规模以上企业40家以上。把民营经济作为县域经济发展的主体力量。坚持大中小并重，引导民营资本拓展经营领域，兴办二三产业，集中扶持一批规模大、实力强、品牌优的民营企业向科技型、外向型、集约型发展。突出抓好民营企业家队伍建设，吸引国内知名民营企业来济宁投资兴业。发挥民营企业信用担保机构的作用，促进民营经济快速健康发展。把增加可用财力作为县域经济发展的重要目标。着力培育发展创税多、贡献大的重点项目、优势企业。加强税收征管，严格控制非税收入非正常增长，不断提高税收收入比重，继续优化财政收入支出结构，争取用好有关政策，逐步实现困难县乡编制内财政供养人员按国家标准发放工资的目标。在壮大经济实力、强化增收节支的基础上，有效化解乡村债务风险。

　　四、有效破解加快发展的瓶颈制约

　　适应国家宏观调控新形势，广开思路，创新办法，有效化解生产要素供应、人才培养引进、生态环境保护、基础设施配套、资源节约利用等方面的矛盾问题，为经济社会全面协调可持续发展提供有力支撑和可靠保障。

　　努力挖掘建设用地潜力。坚持依法用地、节约用地、集约用地、保障用地的原则，编制好新一轮土地利用总体规划，积极预留可发展土地。依法收回长期征而未用、多征少用的土地，最大限度盘活存量土地。落实规划目标，积极争取政策，多方增加投入，加快采煤塌陷地治理步伐，抓好废弃荒地复垦开发，增加耕地保有量。引导园区企业调整建设布局，推行多层厂房，合理确定绿化和道路占地，提高土地利用率。

　　广开渠道筹措建设资金。依托资源优势、产业基础和企业优良资产，吸引国内外大公司、大财团和各类金融机构来我市创业发展，借助外力增加投资。密切银企合作，搞好项目推介，支持商业银行创新金融产品，优化资金投向，持续增加信贷投放。充分利用国内外两个资本市场，精心培植上市资源，着力抓好境外上市，扩大直接融资规模。进一步放宽政策、降低门槛，引导民间资本投向二三产业，提高其在固定资产投资中的比重。发挥政府各类专项资金的吸附引导作用，调动企业自主投资的积极性。

　　加快构筑人才高地。实施人才强市战略，利用多种途径培养急需人才，面向国内外引进外经外贸、城市规划、经营管理、高新技术研发等领域高层次、高技能人才。办好博士后工作站和留学人员、“百名人才”创业园。加强人才市场建设。做好引进国外智力工作。

　　强化生态环境保护。全面落实生态市建设各项任务目标。按照南水北调水质要求，突出抓好水污染防治，深化工业点源治理，组织已达标企业实施“再提高工程”，督促未达标企业高起点完善治污设施，关停治理无望的污染企业。认真执行“三同时”制度，杜绝新污染源产生。有效控制农业面源污染。实施南四湖“碧水行动计划”，强化人工湿地和生态功能保护区建设。大力开展植树造林，重点搞好山区、库区、滩区和沿河、沿路及环城绿化。以小流域综合治理为重点，强化水土保持。构建环境质量监测平台，严厉查处污染环境、破坏生态的违法行为。

　　完善重大基础设施。加快建设50万伏输变电工程。全力支持配合南水北调东线工程和东调南下工程建设。开工建设济（南）菏（泽）高速公路济宁段和327国道济宁城区改线工程。积极争取上级支持，确保济宁始发进京临客列车纳入铁路正图运行。组织专门班子靠上工作，争取增开济宁—上海始发列车、京沪高速铁路在曲阜建站。做好济宁至商丘、济宁至徐州高速公路和京杭运河济宁至东平湖复航工程前期工作。

　　建设资源节约型社会。依法加强监督管理，严格资源开发市场准入，适度控制矿产资源开采总量。制止超层越界、超设计能力的掠夺式开采，提高煤炭企业回采率。以造纸、纺织、酿造、化工、建材等行业为重点，积极推行清洁生产，大力发展循环经济，着力培植菱花集团、雪花集团、太阳纸业等10家循环经济先导示范企业。强化工业“三废”回收和无害化处理，推广农村沼气工程和秸秆综合利用。加快建设济宁垃圾处理厂。强力推行节水器具。增强全社会节约意识，倡导资源节约型生活方式。

　　五、进一步增强城市综合服务功能

　　抓住济宁被列为全省三大都市圈之一的机遇，推动城市规划建设管理再上新水平。

　　城市规划出精品。放开规划建筑设计市场，招标引进国内外一流规划设计力量承担重大规划项目编制任务。继续深化济宁都市圈规划，修编完善城市总体规划，做好市区水系河道、交通干道等专项规划，加快编制市区重点区域和曲阜新区控制性详细规划，形成整体布局和谐统一、功能分区定位准确、单体设计风格独特、城市空间全面覆盖的科学体系。高起点编制县城及镇村规划，促进城乡一体、有机对接。完善城市规划委员会和专家组议事规则，严把规划项目审批关。增强城市规划执法刚性，严肃查处违法违规建设行为。

　　城市建设增亮点。继续推进“绿亮清”工程建设。房地产实行连片综合开发，杜绝零星分散建设。做活水的文章，重点搞好市区古运河沿岸整体规划开发，实施城区河道水系贯通工程，加大北湖景区招商开发力度。做好城北片区综合开发和山推片区等旧城改造项目，建设一批高层组团住宅。完成洸河路西延等城区主干道及小街巷新建、改造任务。抓好亮灯工程延伸配套。实施天然气引进工程，加快推进以供水、供气、供热、排污为重点的城区管网建设。提高施工效率和质量，创出更多一流工程。加快县城基础设施配套建设，扎实推进中心镇建设。

　　城市管理上水平。围绕建立长效机制，健全完善“两级政府、三级管理、四级网络”的城市管理体制。强化城市综合执法功能，严厉查禁乱贴乱画、乱搭乱建行为。取缔非法营运车辆。巩固机动车禁鸣成果。年内完成邹县电厂等18家电厂脱硫改造任务，拆除集中供热范围内小型燃煤锅炉，促使饮食摊点更换洁净能源，二氧化硫低空污染得到有效治理，确保全年城区空气质量优良标准300天以上。加快建设城区雨污分流、截污导流和中水回用工程，已建成的污水处理厂要正常运行。有计划封停城区供水管网内的自备井，确保居民饮水安全。加强市场管理，确保食品、药品安全。

　　城市经营求突破。面向国内外引进有实力的投资者参与城市开发。完善土地收购储备制度，依法处理城市闲置土地。以基础设施先期配套实现土地溢价增值。严格实行经营性建设用地先详规再“招、拍、挂”制度。引入竞争机制推进城市拆迁，降低成本、提高效率。对各类城市资产经营权、冠名权、广告权等实行竞标拍卖，探索重大基础设施市场化运营的有效途径，实现城市资产收益最大化。

　　六、加快构建社会主义和谐社会

　　坚持立党为公、执政为民，围绕社会全面进步和人的全面发展，协调推进“三个文明”建设，切实维护人民群众政治、经济、文化权益，让广大城乡居民安居乐业，共享改革发展成果。

　　努力提高人民生活水平。严格执行最低工资规定，查处和纠正拖欠克扣职工、农民工工资行为。加快发展扩大就业容量，落实再就业政策，完善就业服务体系，有效解决停产、破产企业职工再就业问题。全年新增城镇就业9万人。完善企业养老、失业保险制度，加快推行基本医疗保险、工伤保险，依法强化社会保险扩面征缴和劳动保障监察执法。规范城乡最低生活保障制度，增加各级财政低保投入。搞好经济适用房和廉租房建设，解决中低收入家庭住房困难。发展社会慈善事业。做好困难群众生活救济工作。年内基本实现“村村通油路”目标。强化农村初级卫生保健，继续推进改水改厕，扩大新型合作医疗试点范围，建立贫困农民医疗救助制度。及时改造修缮中小学危房，努力降低辍学率，继续实施“万名特困生救助工程”。做好农村养老保险工作，健全农村“五保”“四级联保”机制。探索建立失地农民社会保障制度。年内继续办好10件实事。

　　深入实施科教兴市战略。健全科技创新体系，支持大中企业高起点建设研发机构，新建5个省级工程技术中心，争取100项攻关项目列入省以上科技创新计划。密切与大专院校、科研院所联系，继续开展“院士济宁行”活动，充分利用科教资源，推进产学研结合。做好专利申请、实施与转化工作，加强知识产权保护。加强科普宣传，倡导移风易俗。完善以县为主的义务教育管理体制，严格实行义务教育“一费制”，巩固提高“普九”水平。实施“名校扩张”战略，年内创建10所省级规范化高中。搞好职业技术教育资源整合，合理调整学校布局和专业、课程设置，探索集团化、连锁式办学新路子。完善济宁学院办学条件，做好济宁师专晋升本科申报工作。支持曲阜师范大学、济宁医学院立足济宁加快发展。

　　扎实推进文明城市创建。加强全民思想道德教育，广泛开展群众性精神文明创建活动，深化“农村文明一条街”建设。深入实施“文化名市建设纲要”，配合省和国家做好“中华文化标志城”前期工作。强化文化市场监管，发展文化产业。加快新闻出版、广播影视和档案、史志、文博等事业发展。年内完成疾病防控体系和突发公共卫生事件医疗救治体系建设任务，改善56处中心镇卫生院医疗条件，发展社区卫生服务，推进中医药现代化进程。积极应对第四次人口出生高峰，强化人口和计划生育基层基础工作，倡导晚婚晚育，重视解决出生婴儿性别比问题，实现稳定低生育水平目标。认真做好妇女、儿童、老龄、残疾人和红十字会工作。深入开展全民健身活动，积极备战21届省运会。

　　加快依法治市进程。认真执行市人大及其常委会决议决定，及时报告政府工作，自觉接受人大监督。积极支持人民政协履行职责，主动听取各民主党派、工商联、无党派人士和各人民团体意见建议，认真办理人大代表议案建议和政协委员提案。做好第八届村（居）委会换届选举工作。完成“四五”普法任务。全面落实“平安济宁”建设各项措施，开展经常性严打整治斗争，严厉打击各种刑事犯罪和黑恶势力，增强社会治安防控能力。做好人民来信来访工作，建立矛盾纠纷排查调处机制，完善各类突发事件应急处置预案。重视国家安全工作。加强国防教育和民兵预备役建设，巩固发展双拥共建成果。重视民族、宗教和外事、侨务、对台工作。落实安全生产责任制，严防各类重大恶性事故发生。做好人防、气象、地震工作，增强防灾减灾能力。

　　各位代表！编制济宁市国民经济和社会发展第十一个五年规划和2020年远景目标，是今年的一项重要工作。我们将按照中央、省和市委的部署要求，以科学发展观为指导，在深入开展调研论证、广泛征求意见建议的基础上，科学确定“十一五”规划和2020年远景目标的指导方针、战略重点和主要任务，力争编制出一个宏观性、前瞻性与指导性、可操作性有机统一的好规划。

　　七、切实加强政府执政能力建设

　　新形势新任务对政府工作提出了新的更高要求。各级政府和公务员要以开展保持共产党员先进性教育活动为动力，加强学习、增长才干，把握大局、多谋善断，求真务实、开拓创新，严格自律、廉洁勤政，不断提高执政能力和领导水平，真正做到想干事、会干事、干成事。

　　建设责任政府。以科学发展观和正确政绩观为指导，认真履行经济调节、市场监管、社会管理和公共服务职能。完善群众参与、专家咨询与集体决策相结合的决策机制，提高政府决策的科学化、民主化水平。对年度任务目标、重大决策部署层层分解责任，确保落实到位。坚持对上负责与对下负责相统一，既要确保政令畅通，又要恪尽为民之责，真心实意为人民排忧难、办实事、谋利益。坚持当前负责与长远负责相统一，既要确保经济社会发展不断上新台阶，又要为长远发展夯实基础、积蓄后劲，创造经得起实践、群众和历史检验的政绩。

　　建设法治政府。贯彻落实《全面推进依法行政实施纲要》，严格按照法定权限和程序行使职责权力。认真执行《行政许可法》，提高审批效能和服务质量。建设高素质行政执法队伍，确保严格执法、公正执法、文明执法。强化行政监督，做好行政复议工作，真正把有权必有责、用权受监督、侵权须赔偿、违法受追究的原则落到实处。

　　建设阳光政府。全面推行政务公开，切实保障广大人民群众的知情权、参与权和监督权。建立完善政府新闻发言人制度。大力推进电子政务，构建开放式政府信息平台。进一步办好市长公开电话和市长信箱，及时倾听群众呼声，改进政府工作。

　　建设清廉政府。牢记“两个务必”，坚持从严治政，筑牢拒腐防变的思想道德防线。认真执行廉洁自律各项规定和全市领导干部“十个不许”廉政纪律，全面落实党风廉政建设责任制，切实规范从政行为。坚决纠正损害群众利益的不正之风，严厉查禁“三乱”行为。全面推行政府采购和“收支两条线”制度，努力从源头上预防和治理腐败。加强领导干部经济责任审计，支持纪检监察机关依法查办案件，严惩腐败分子。

　　建设务实政府。坚持说实话、出实招、办实事、求实效，力戒形式主义，坚决反对弄虚作假、欺上瞒下。提高工作标准，以只争朝夕的精神，赶超先进目标，争创一流业绩，防止和克服推诿扯皮、无所作为的不良风气。从市政府做起，进一步压缩文件、减少会议，集中精力抓落实。倡导敢抓敢管、敢于负责、敢于碰硬，深入一线、亲历亲为，努力提高解难题、化矛盾、破瓶颈、谋发展的能力。

　　各位代表！孔孟之乡这一方圣地沃土，正焕发出蓬勃生机和旺盛活力。机遇前所未有，发展时不我待。让我们在中共济宁市委的坚强领导下，以饱满的热情、昂扬的斗志、创新的精神、务实的作风，奋力夺取改革开放和现代化建设新胜利，为建设经济强市、文化名市、组群结构大城市做出新的更大贡献！
